# Fludarabine, adriamycin and dexamethasone (FAD) in newly diagnosed advanced follicular lymphoma: a phase II study by the British National Lymphoma Investigation (BNLI)

**DOI:** 10.1038/sj.bjc.6602031

**Published:** 2004-07-27

**Authors:** L Yung, D Cunningham, B Hancock, P Smith, K Maclennan, D Linch, A McMillan

**Affiliations:** 1 Department of Haematology, University College London, UK; 2 Department of Oncology, Royal Marsden Hospital, Sutton, UK; 3 Department of Oncology, Royal Hallamshire Hospital, Sheffield, UK; 4 British National Lymphoma Investigation, 222 Euston Road, London, UK; 5 Department of Histopathology, University of Leeds, Leeds, UK; 6 Department of Haematology, Nottingham City Hospital, Nottingham, UK

**Keywords:** chemotherapy, fludarabine, follicular, lymphoma

## Abstract

The optimal first-line treatment for symptomatic patients with advanced stage follicular lymphoma remains unclear. Fludarabine-based combination regimens have been extensively used in relapsed disease and merit consideration as first-line therapy. We here report the results of a phase II study of FAD (fludarabine, adriamycin, dexamethasone) regimen in 30 patients with advanced stage follicular lymphoma requiring treatment. The response rate was in excess of 90% with 39% achieving a complete remission. The major toxicity was myelosuppression, but only 3% of cycles were associated with grade IV leucopenia. The high response rate has not translated into major improvements in failure-free survival and consideration must be given to alternative treatment modalities to consolidate the high rate of initial responses.

Follicular lymphoma remains a disease of relapses and remissions, and despite new modalities of therapy a plateau in the survival curve has yet to become apparent. Treatment with the alkylating agents chlorambucil and cyclophosphamide have been the mainstay of therapy for many years. Nucleoside analogues such as fludarabine offer an alternative, noncrossresistant option. Fludarabine has been shown to be effective in low-grade non-Hodgkin's lymphomas although results are disappointing when it has been used as single agent first-line therapy (Solal-Celigny *et al*, 1996). In an Intergroup randomised trial, fludarabine resulted in a better response rate than CVP (cyclophosphamide, vincristine and prednisolone), but this did not translate into improved time to treatment failure or overall survival (OS) (Marcus *et al*, 2002). McLaughlin *et al* reported good results using fludarabine in combination with mitoxantrone and dexamethasone (FMD/FND) in patients with recurrent disease. The phase I study in 21 patients receiving up to eight courses of FMD reported a 43% complete remission (CR) rate with these remissions lasting a median of 18 months (McLaughlin *et al*, 1994). These results were confirmed in the phase II study of 51 patients with recurrent or refractory indolent lymphoma, which reported a response rate of 98%, with attainment of CR in 47% (McLaughlin *et al*, 1996). Another phase II study evaluated FMD in 54 mainly pretreated patients, and reported an overall response rate of 69%, with complete responses seen in 11 (20%) patients (Crawley *et al*, 2000). Treatment toxicity was not uncommon with these combination regimens. In the McLaughlin study, there were six recorded cases of Herpes zoster infection and six cases of proven or suspected *Pneumocystis carinii* in 257 courses of treatment. As a result of these opportunistic infections, cotrimoxazole prophylaxis was introduced towards the end of the trial period (McLaughlin *et al*, 1996). In the Crawley study, chemotherapy was delayed in five and discontinued in a further five patients due to prolonged cytopenia. A total of 18 patients required inpatient admission for treatment of neutropenic fever (Crawley *et al*, 2000).

There is relatively little information about fludarabine, anthracyline/anthracenedione and steroid-containing combination chemotherapy regimens as first-line therapy. The patients in the McLaughlin study were all pretreated and there were only 10 chemotherapy naive patients in the Crawley study. Velasquez *et al* (2003) and Zinzani *et al* (2000) conducted studies in previously untreated patients with 78 and 27 patients, respectively, but they used fludarabine and mitoxantrone only, omitting the steroids which can have potent lympholytic activity (Cline, 1973; Greenstein *et al*, 2002).

There has been controversy about the relative merits of mitoxantrone and adriamycin in the treatment of lymphomas (Sonneveld *et al*, 1995; Mainwaring *et al*, 2001; Osby *et al*, 2003), and in this trial adriamycin was used instead of mitoxantrone in view of the lesser myelosuppression reported by Mainwaring *et al* (2001) at the doses used.

## PATIENTS AND METHODS

Patients over the age of 18 years with newly presenting follicular non-Hodgkin's lymphoma, types B–D according to the Working Formulation, with stage III–IV disease requiring therapy were eligible for this trial.

Atotal of 35 patients were entered, with a median age of 55 years (range 29–76). There were 21 (60%) male and 14 (40%) female patients. All histopathology specimens were centrally reviewed, and after such review five patients were excluded (three mantle cell lymphomas, one large cell lymphoma, one insufficient material). This report is based on the 30 eligible patients with a confirmed diagnosis of follicular lymphoma. In all, 16 (46%) patients had stage III disease and 19 (54%) had stage IV disease. All were judged as requiring treatment (progressive disease over at least 3 months, critical organ involvement, bone marrow failure or ‘B’ symptoms.) A total of 19 patients (58%) had WHO performance status of 0.

Patients were excluded from this study if they were suffering from any irreversible medical condition that was likely to be fatal within the next 5 years or which would preclude the use of combination chemotherapy. Patients with impaired renal function (creatinine >150 gmol l^−1^), hepatic impairment (bilirubin >35 gmol l^−1^) or cardiac dysfunction (previous history and MUGA <40%) not due to involvement with follicular lymphoma were excluded from this study. Other exclusion criteria were a diagnosis of a previous malignancy or non-Hodgkin's lymphoma affecting the central nervous system.

The patient characteristics are shown in Table 1

**Table 1:** Patient characteristics

	**Eligible patients (*n*=30)**	
*Sex*	*n*	%
Male	16	53
Female	14	47
		
*Criteria for treatment*		
Progressive disease	13	43
Critical organ failure	9	30
Symptoms	16	53
		
*Age*		
Median	55	
Range	29–76	
		
*Stage*		
III	14	47
IV	16	53
		
*B symptoms*	17	57
		
*WHO PS*		
0	19	63
1	7	23
2	3	10
3	1	3
		
*IPI*		
Low	0	
Low–intermediate	17	57
Intermediate–high	9	30
High	3	10
Unknown	1	3

.

### Trial design

This was a single arm study of the toxicity and efficacy of the nucleoside analogue fludarabine monophosphate in combination with adriamycin and dexamethasone (FAD). The primary end point was regimen-related toxicity with a secondary end point of failure-free survival (FFS).

The treatment schedule of ‘FAD’ comprised fludarabine 25 mg m^−2^ intravenously on days 1–3, adriamycin 50 mg m^−2^ intravenously on day 1, and dexamethasone 20 mg orally or intravenously from days 1–5. Co-trimoxazole, 480 mg b.i.d. thrice weekly throughout treatment and for 8 weeks following completion of therapy, was given as prophylaxis against *Pneumocystis carinii* infection. Allopurinol 300 mg once daily orally was also administered for the first week of each course of chemotherapy. Courses were given on a 28-day cycle until CR was achieved, with two courses given beyond remission as remission consolidation. Thus, patients received a minimum of four and a maximum of eight cycles of chemotherapy.

All blood products required were irradiated to a minimum of 25 Gy. Any significant pleural effusions or abdominal ascites were drained to dryness prior to commencement of fludarabine therapy. The first course of chemotherapy was given at full dose. In the event of haematological toxicity, dose modifications for subsequent courses were made as follows: treatment was deferred if the neutrophil level was <1.5 × 10^9^ l^−1^ or platelet count <100 × 10^9^ l^−1^. If after a week's delay, the threshold levels were still not reached, the full blood count was taken again and treatment was delayed a further week. If after 2 weeks delay the threshold was not reached, the following dose adjustments were made:


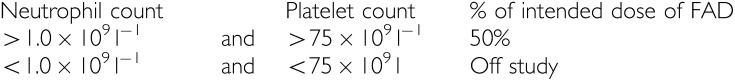


If either the platelet count or the neutrophil levels were below the threshold levels, then the lower dose was to be given.

For hepatic or renal impairment, treatment was delayed for 2 weeks until the abnormal result fell within the laboratory normal range. If after this period the liver or renal function was still abnormal, the patient was withdrawn from the study. Patients were withdrawn from the study if neurological toxicity was greater than WHO grade II.

No maintenance therapy was given as part of this study.

### Response evaluation

Patients were clinically assessed after two cycles of therapy. Those with clinically apparent progressive disease (PD) or with no response after two cycles were withdrawn from the study. The remaining patients were assessed after four cycles with CT scanning. Those in CR or with good partial response (PR) (>75% tumour reduction on CT) received a further two courses of treatment. Those with a maximum partial response (>50% tumour reduction) received two further courses from maximal partial response or a total of eight cycles (whichever was the least).

### Statistical considerations

The primary end point of the study was regimen-related toxicity using toxic death rate and toxicity as outcome measures. The secondary end point was FFS using CR rate, progression-free survival, disease-free survival and OS as outcome measures. The survival analyses are presented as Kaplan–Meier survival graphs.

## RESULTS

### Response rates

The overall response rate in this cohort of patients was 96% with a CR rate of 39%. If the two patients dying of myocardial infarction are considered as nonresponders, the overall response rate is 93%.

**Figure 1:**
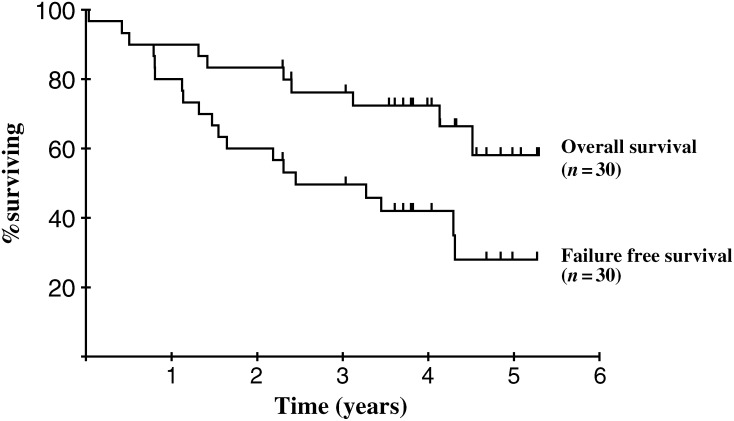
Overall survival and failure-free survival.

With a median follow-up of 50 months (range 839–1932 days) in the 20 patients who are still alive, the actuarial 4-year OS rate is 72% (Figure 1

).

In all, 50% of patients have experienced disease progression at some time during the period of this study. The median time to treatment failure was 29 months and the actuarial 4-year FFS is 42% (Figure 1).

### Toxicity

Haematological toxicity was mild. No significant anaemia was reported for any of the 181 courses of chemotherapy administered to patients in this study. In total, 23% of courses of FAD chemotherapy resulted in significant leucopenia (WHO grade III/IV). Severe thrombocytopenia was reported in one course only.

Nonhaematological toxicity was rare, with 3% of courses causing grade III nausea and vomiting, and 7% of patients reporting grade III alopecia. Mucositis was mild, with grade I or II symptoms reported in 17 courses of chemotherapy.

### Deaths

There were a total of 10 deaths in this cohort of 30 patients. Seven were due to non-Hodgkin's lymphoma. Two patients died of myocardial infarction, one occurring 1 week after the first course of FAD chemotherapy, and the other occurring 3 weeks after the sixth course. One patient developed an ischiorectal abscess after his second course of FAD chemotherapy, which required formal surgical debridement and a prolonged inpatient stay of several months. He eventually died of a bronchopneumonia 6 months after starting treatment (Tables 2

**Table 2:** Treatment toxicity: haematological (a), non-haematological (b)

	**Courses for eligible patients (*n*=181)**	
	***n***	**%**
(a) Haematological toxicity		
*Hb*		
0	147	81
1	24	13
2	1	0.6
3		
4		
Unknown	9	5
		
*WBC*		
0	92	51
1	22	12
2	16	9
3	36	20
4	6	3
Unknown	9	5
		
*Platelets*		
0	168	93
1	3	1.5
2		
3	1	0.6
4		
Unknown	19	10
		
(b) Nonhaematological toxicity		
*Oral*		
0	146	80
1	6	3
2	11	6
3		
4		
Unknown	18	10
		
*Nausea and vomiting*		
0	132	73
1	19	10
2	7	4
3	5	3
4		
Unknown	18	10
		
*Diarrhoea*		
0	162	90
1	6	3
2	3	1.5
3		
4		
Unknown	10	5.5
		
*Neurological*		
0	158	87
1	13	7
2		
3		
4		
Unknown	10	5.5
		
*Skin*		
0	164	90
1	1	0.6
2		
3		
4		
Unknown	16	8.8
		
*Alopecia*		
0	45	25
1	32	18
2	46	25
3	13	7
4		
Unknown	45	25

, 3

**Table 3:** Response rates

	***N*=30**	**%**
*Response*	30	
CR	11	37
PR	16	53
PD	1	3.3
Not evaluable^a^ (see footnote)	2	6.6
		
*Disease progression*	15	50
		
*Status*		
Alive	20	67
Dead	10	33

a These two patients died of myocardial infarction, one occurring 1 week after the first course of chemotherapy and the other 3 weeks after the sixth course. The response to chemotherapy was therefore not evaluated.

and 4

**Table 4:** Deaths

**Cause of death**	***N*=30**
NHL	7
Cardiovascular disease	2
Bronchopneumonia	1

).

## DISCUSSION

This study confirms that a combination of fludarabine, adriamycin and dexamethasone can be administered with acceptable toxicity to patients with follicular lymphoma, many of whom are over 60 years of age. The prophylactic use of cotrimoxazole successfully prevented the development of *Pneumocystis carinii* infection without obvious increased haematological toxicity. Only 3% of cycles resulted in grade 4 neutropenia, and significant thrombocytopenia was even more rare. Nonhaematological toxicity was also very rare except that two patients suffered fatal myocardial infarctions to which the adriamycin might have contributed.

The overall response rate in these previously untreated patients was very high (93%) and accords with the results reported by Velasquez *et al* (2003) and Zinzani *et al* (2000). There is no major difference in efficacy between the use of adriamycin as in this study or the use of mitoxantrone as in the regimen developed by McLaughlin *et al* (1994; 1996), but only a large randomised trial would detect small differences.

The response rates to both fludarabine and mitoxantrone (Zinzani *et al*, 2000; Velasquez *et al*, 2003) and to fludarabine, mitoxantrone and dexamethasone as reported here are markedly higher than those reported with fludarabine alone. In a phase II trial of 54 patients reported by Solal-Celigny *et al* (1996), the overall response rate to fludarabine was 65%, and in the Intergroup randomised trial comparing CVP to fludarabine, the overall response to fludarabine was 69% (Marcus *et al*, 2002).

Unfortunately, the higher response rate in this study has not translated into prolonged remissions. The median time to treatment failure of 29 months is only marginally greater than the 21 months seen in the Intergroup study with fludarabine alone.

However, if prolonged remissions are to be achieved in a large proportion of patients with follicular lymphoma, then high initial responses must first be achieved and the combination of FAD or FMD are probably the most effective chemotherapy regimens, with acceptable toxicity, described to date. Additional agents are required to either consolidate or maintain such responses and rituximab combined with these regimens merits consideration as first-line therapy.
